# Consumption of glucose drinks slows sensorimotor processing: double-blind placebo-controlled studies with the Eriksen flanker task

**DOI:** 10.3389/fnhum.2013.00651

**Published:** 2013-10-24

**Authors:** Christopher Hope, Ellen Seiss, Philip J. A. Dean, Katie E. M. Williams, Annette Sterr

**Affiliations:** School of Psychology, University of SurreyGuildford, Surrey, UK

**Keywords:** blood glucose concentration, performance, reaction time slowing, cognition, euglycemia, humans, cognitive control, conflict monitoring

## Abstract

Modulations of blood glucose concentration (BGC) in the normal range are known to facilitate performance in memory and other cognitive tasks but few studies have investigated the effects of BGC variations on complex sensorimotor task so far. The present study aimed to examine glucose effects with the Eriksen flanker task. This task was chosen because it can dissociate between the effects of BGC on sensorimotor processing and cognitive control by assessing congruency effects. In two linked double-blind placebo-controlled experiments BGC was elevated within the normal BGC range (4–7 mmol/l) by approx. 1.5 mmol/l with glucose drinks and compared to a placebo drink condition while a flanker task with either strong or weak stimulus-response (SR) mapping was performed. Modulation of the performance in the flanker task by glucose was linked to the strength of the SR mapping but not congruency effects. Under weak SR mapping, reaction times (RTs) were slowed in the glucose condition compared to placebo while error rates remained unchanged, whereas cognitive control was not affected by glucose. When SR mapping was strong, no differences were found between glucose and placebo. Enhanced glucose levels differentially affect behavior. Whereas the literature mainly reports facilitating characteristics of enhanced glucose levels in the normal range, the present study shows that higher glucose levels can slow RTs. This suggests that glucose does not have a uniform effect on cognition and that it might be differential depending on the cognitive domain.

## INTRODUCTION

Teachers and parents frequently report that the consumption of glucose-rich foods is detrimental to children’s classroom behavior and their learning. Moreover, a sugar ban in an American school is reported to have led to a fall in disciplinary incidents, counseling referrals and an increase in exam performance and reading scores (Park, 2008; Radnedge, 2009). To the contrary, advertisements for energy drinks suggest that sugary drinks can enhance cognitive performance in stressful situations such as exams or before physical activities by giving our body and brain more energy. Given that the interaction of glucose consumption with cognition and behavior is potentially hugely important, further research in this area is clearly needed.

Reviews suggest that experimental elevation of blood glucose concentration (BGC) levels in the euglycemic range (4–7 mmol/l) typically facilitates performance in cognitive tasks (Riby, 2004; Feldman and Barshi, 2007). This is particularly true for studies on declarative memory and working memory, as well as tasks with high cognitive demands (Riby, 2004; Feldman and Barshi, 2007; Smith et al., 2011). However, only some studies investigated experimentally elevated BGC on sensorimotor tasks (e.g., Donohoe and Benton, 2000; Scholey et al., 2009), and even fewer studies examined glucose effects on conflict tasks. More specifically, only four studies investigated glucose drink effects using the Stroop task (Benton et al., 1994; Craft et al., 1994; Gailliot et al., 2007; Brown and Riby, 2013), and these did not show a coherent picture: Craft et al. (1994) and Benton et al. (1994) found a glucose facilitation effect for reaction times (RTs), Brown and Riby (2013) reported a borderline significant glucose facilitation effect on RTs, and Gailliot et al. (2007) found no glucose effects. These discrepant findings might be explained by methodological differences between the studies, such as fasting instructions or the control of BGC prior to and/or throughout the experiment. Alternatively, it might be the case that conflict tasks are less susceptible to glucose effects.

The present study therefore aimed to further investigate the effect of glucose on conflict tasks. For this purpose we examined the effects of glucose-enriched drinks with the Eriksen flanker task (Eriksen and Eriksen, 1974) in two double-blind placebo-controlled studies. The use of the flanker task enabled us to explore if glucose effects can be found in a wider range of conflict tasks beyond the Stroop task. The task was chosen because of its experimental robustness, and its ability to separately assess stimulus-response (SR) mapping strength and cognitive control, reflected in the size of the congruency effect. In the flanker task, participants respond to a central target stimulus (e.g., left or right-pointing arrow) by pressing the corresponding response key (left vs. right key). This central target stimulus is flanked by stimuli that are either congruent (e.g., flanker arrows point in the same direction as the target arrow), neutral (e.g., flankers are horizontal bars), or incongruent (e.g., flanker arrows and target arrow point in the opposite direction), which are not relevant for the participants response. Typically, the flankers automatically activate a response tendency, which either corresponds to the response tendency elicited by the target (congruent trials) or is in conflict with it (incongruent trials). This response conflict in the incongruent condition results in RT costs and increased error rates when compared to the other conditions (Gratton et al., 1992; Ridderinkhof et al., 1995).

In the present study, we used a double-blind placebo-controlled study design in which BGC levels were experimentally modulated to be at the upper end of the euglycemic range (4–7 mmol/l) for the period of the experiment in the glucose condition, and at the lower end of the euglycemic range in the placebo condition. This was achieved through a protocol which controlled baseline BGC through overnight fasting and subsequent standardized breakfast followed by a schedule of glucose drinks to produce a steady elevation in BGC relative to the placebo drinks during task performance. Two experiments were conducted. Experiment 1 employed a simple version of the flanker task using arrow stimuli that induced strong SR mapping. Experiment 2 used a more demanding version of the task with letter stimuli and changing SR sets from block to block. This required frequent formation of novel SR mapping rules and hence a weaker SR binding throughout the experiment, resulting in higher task demands. In both studies, we measured congruency effects, i.e., the difference between congruent and incongruent trials. Based on the literature suggesting a relationship between high task demands and the magnitude of performance modulation by glucose (Riby, 2004), we predicted stronger glucose effects on SR mapping and congruency in experiment 2.

## EXPERIMENT 1

Experiment 1 used an arrow version of the Eriksen flanker task (Eriksen and Eriksen, 1974) with congruent, incongruent and neutral flankers. Performance in this task was compared between the placebo condition and an intervention condition in which glucose levels were elevated to the upper normal range through the consumption of glucose drinks. For both conditions, we expected to find the typical flanker effects with congruent trials being faster and more accurate than incongruent trials. Based on previous studies (Craft et al., 1994; Brown and Riby, 2013) we also predicted that glucose may speed up RTs, especially in incongruent trials, maybe at the cost of producing more errors on these trials.

### METHODS (EXPERIMENT 1)

#### Participants

Twelve participants (mean age: 25.1 ± 2.1 years; 6 females) took part in the study. None of the participants were diabetic or had any other glucoregulatory problems as assessed by self-report. This experiment was given a favorable opinion by the University of Surrey Ethics Committee. All participants gave written informed consent and were paid £7.50 per hour.**

#### Design

A double-blind placebo-controlled within participants design was used in which each participant was tested in two sessions, receiving glucose drinks in one session and placebo drinks in the other session. The order of sessions was counterbalanced across participants. Experimenter and participant were blind to the content of the drinks and to the BGC measurements taken during the study.

#### Materials

***Breakfast.*** Participants were provided with a standardized breakfast similar to the one used by (Sünram-Lea et al., 2001). It consisted of 1 toasted New York Bakery Co. Plain Bagel®, Tesco Value Soft Cheese® spread thinly over the bagel, and a 150 g Yeo Valley Organic Yogurt®.

***Glucose and placebo drinks.*** Each drink consisted of 100 ml of water and 100 ml of freshly squeezed lemon juice to disguise the taste difference between glucose and placebo drinks. Glucose drinks also contained 25 g of glucose powder whereas placebo drinks instead contained 2 mg of saccharin. Extensive pilot testing was conducted to ensure that the two drink types could not be distinguished by taste.

***Blood glucose concentration measurements.*** Blood samples were obtained using disposable Unistick 3 Comfort (Owen Mumford, Ltd.) safety lancets. The BGC of these samples was assessed using an Ascensia Contour blood glucose meter (Ascensia Ltd.).

#### Flanker task

**FIGURE 1:**
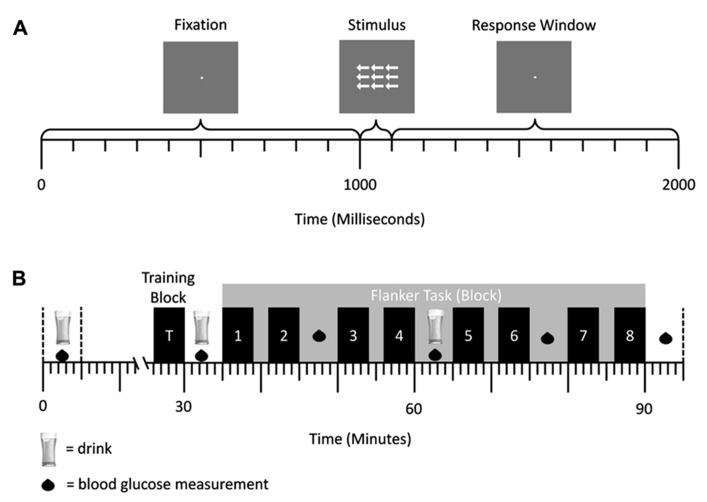
**(A)** Trial structure of the flanker task in experiment 1. A central fixation dot was presented for 1000 ms, followed by an arrow array for 100 ms. Afterward the central fixation dot was presented again for 900 ms. Responses could be conducted after stimulus onset and before the onset of the first fixation cross (response window = 1000 ms). **(B)** Schematic representation of a testing session. Glucose/placebo drinks were administered at 30 min intervals, while BGC measurements were taken every 15 min. The eight blocks of the flanker task were performed after administration of the second drink. They are represented by the numbered bars.

**Figure 1A** shows a schematic illustration of the trial structure. Trials started with the presentation of a fixation dot for 1000 ms. This was followed by an array of arrows for 100 ms and then the presentation of a fixation dot in the center of the screen for 900 ms, in which time a response could be made. Stimuli were white presented against a medium gray background. Arrow arrays consisted of a central target arrow, pointing either left or right, surrounded by flanking arrows. Participants were required to press a response button on their right side of space with their right hand if a right pointing central target arrow was presented, or press a button on their left side of space with their left hand if a left pointing central target arrow was presented as fast and as accurately as possible. Flanking stimuli were either congruent (i.e., pointed in the same direction as the target arrow), incongruent (i.e., pointed in the opposite direction to the target arrow), or neutral (arrow stems with no head) relative to the target response. The size of the array of arrows was 1.3° × 2.3°. Individual arrows were 0.7° long, and had a stem 0.1° wide. The arrow heads were 0.2° long and 0.3° wide. Neutral flankers were 0.7° long, and had a stem 0.1° wide.**Participants were positioned 85 cm from the screen. The task consisted of eight blocks with 120 trials, with a mandatory 2 min break between blocks (see **Figure 1B**). Within each block, 40 trials of each of the three trials types (congruent, neutral, and incongruent) were presented in a random order.

#### Procedure

The experimental protocol is summarized in **Figure 1B**. To control for initial BGC at the start of the experiment, participants were asked to fast from midnight prior to the study and eat a standardzsed breakfast at 8.00 am prior to testing which was provided to them free of charge. In addition they were allowed to drink water if they became thirsty. Upon arrival at the laboratory at 9.30 am participants signed a consent form and then the experimenters setup EEG equipment to record physiological data, taking around 60 min^1^. At ~10.30 am a blood glucose measurement was taken, if the reading was ≤5.5 mmol/l the first drink was administered. Further BGC measurements were taken in 15 min intervals, and two more drinks were administered at 30 min intervals in order to ensure a constant BGC level throughout the experimental task. When an initial BGC reading was >5.5 mmol/l, additional readings were taken in 15 min intervals until a BGC ≤5.5 mmol/l was reached. After the first drink, participants were given the Edinburgh Handedness Inventory (Oldfield, 1971) and they performed a training block of the task consisting of 30 trials. A second drink was given 30 min after the first one. Immediately afterward, the flanker experiment started and lasted for approximately 1 h.

#### Data analysis

Blood glucose concentrations were analyzed by comparing the baseline difference between the glucose and placebo condition (1st BGC measurement) with a paired samples *t*-test. Drink effects on the BGC during the performance of the flanker task were assessed by a repeated-measures ANOVA with the factors DRINK TYPE (glucose vs. placebo) and BGC TIME (3rd–7th BGC measurements). RTs and choice error rates served as behavioral measures for the flanker task. Trials with early (RT < 100 ms), late (RT > 1000 ms), or missed responses, and choice errors were discarded from the RT analysis. RTs and choice error rates were separately analyzed in a mixed four-way ANOVAs comprising the within-participant factors DRINK TYPE (glucose vs. placebo), TRIAL TYPE (congruent vs. neutral vs. incongruent), BLOCK (1–8), and the between-participant factor DRINK ORDER (placebo 1st/glucose 2nd, vs. glucose 1st/placebo 2nd). For all ANOVAs, violations of sphericity were corrected using the Huynh–Feldt correction. *Post hoc* *t*-tests were conducted and Bonferroni correctioned as appropriate.

### RESULTS (EXPERIMENT 1)

#### BGC measurements

**FIGURE 2:**
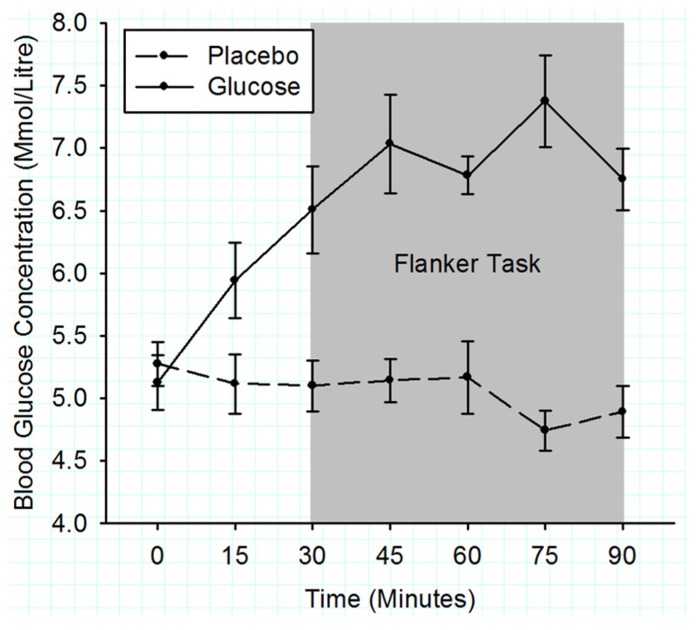
**Mean blood glucose concentrations measured in 15 min intervals for the duration of experiment 1.** Solid lines represent the BGC in the glucose condition and dashed lines the BGC in the placebo condition. The gray shaded area indicates the time period when the flanker task was performed. Error bars represent the standard error of the mean.

**Figure 2** shows that the initial BGC levels were very similar for both drink types (placebo: 5.1 ± 0.2 mmol/l; glucose: 5.3 ± 0.2 mmol/l; *t* (11) = 1.17, *p* = 0.27). During the flanker task (3rd–7th BGC measurement), the BGC was significantly elevated in the glucose compared to the placebo condition (6.9 ± 0.2 vs. 5.0 ± 0.2 mmol/l), *F* (1, 10) = 78.12, *p* < 0.001.

#### Behavioral measures

**FIGURE 3:**
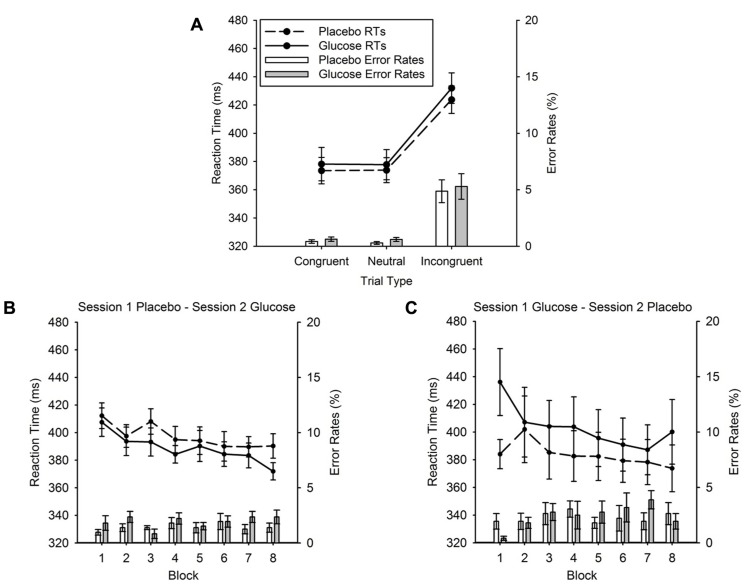
**(A)** Mean reaction times (lines) and error rates (bars) for the congruent, neutral and incongruent conditions of the flanker task for glucose (solid lines; gray bars) and placebo (dashed lines, white bars) in experiment 1. **(B)** Session effects in the task, session 1 – placebo, session 2 – glucose. Mean reaction times (lines) and error rates (bars) for glucose (solid lines; gray bars) and placebo (dashed lines, white bars) and for each block separately. **(C)** Session effects in the task, session 1 – glucose, session 2 – placebo. Mean reaction times (lines) and error rates (bars) for glucose (solid lines; gray bars) and placebo (dashed lines, white bars) and for each block separately. Error bars represent the standard error of the mean.

***Reaction times.*** **Figure 3A** shows a clear flanker congruency effect. Responses in incongruent trials were slower than responses in congruent and neutral trials as indicated by a significant main effect of TRIAL TYPE, *F* (2, 20) = 259.35, *p* < 0.001. *Post hoc* tests showed that RTs in incongruent trials (428 ± 10 ms) were significantly slower compared to RTs in congruent trials (376 ± 10 ms; *t* (11) = 5.56, *p* < 0.001) and neutral trials (376 ± 9ms; *t* (11) = 6.77, *p* < 0.001). More importantly, the drink type did not affect the RTs, as there was no significant main effect of DRINK TYPE, *F* < 1, and no interaction between DRINK TYPE and TRIAL TYPE, *F* = 1.

Figures 3B and 3C display the decrease of RTs over the successive blocks of the experiment. This was confirmed statistically with a main effect of BLOCK, *F* (7, 70) = 11.47, *p* < 0.001. Furthermore, when glucose was administered in session 1 and placebo in session 2 (**Figure 3C**), RTs recorded in block 1 were slowed by glucose administration compared to placebo. This effect was not found for later blocks and it was not present when placebo was administered in session 1 and glucose in session 2 (**Figure 3B**). This observation was confirmed with a significant DRINK TYPE × DRINK ORDER × BLOCK interaction, *F* (7, 70) = 2.17, *p* = 0.047. To explore this interaction further, we conducted a *post hoc* analysis of block 1 using a 2 × 2 mixed ANOVA with the factors DRINK TYPE and DRINK ORDER. This ANOVA revealed a significant main effect of DRINK TYPE, *F* (1, 10) = 6.71, *p* = 0.03, and a highly significant DRINK TYPE × DRINK ORDER interaction, *F* (1, 10) = 9.65, *p* = 0.01, caused by a significant response slowing for glucose compared to placebo (glucose = 436 ± 24 ms, placebo = 384 ± 26 ms; *t *(5) = 3.32, *p* = 0.021) when glucose was administered in the first and placebo in the second session. However, this slowing was absent when placebo was administered in the first and glucose in the second session (glucose = 407 ± 10 ms, placebo = 412 ± 9 ms, *t* (5) = 0.50, *p* = 0.64).

***Error rates.*** **Figure 3A** displays a clear flanker congruency effect for error rates. This main effect of TRIAL TYPE was significant, *F* (2, 20) = 21.27, *p* < 0.001. Participants made more errors in the incongruent (5.08 ± 0.98%) compared to the congruent (0.52 ± 0.13%; *t* (11) = 4.223, *p* = 0.001) and neutral condition (0.44 ± 0.13%; *t* (11) = 4.413, *p* = 0.001). Similar to the RTs, there was no main effect of DRINK TYPE, *F* (1, 10) = 1.92, *p* = 0.20, and no DRINK × TRIAL TYPE interaction, *F* < 1. In contrast to the RTs, error rates remained constant across successive blocks of the task (Figures 3B and 3C), as reflected in the non-significant main effect of BLOCK, *F* (7, 70) = 1.68, *p* = 0.13.

### DISCUSSION (EXPERIMENT 1)

With experiment 1 we tested the hypothesis that performance in the Eriksen flanker task (Eriksen and Eriksen, 1974) would differ between glucose and placebo. More specifically, we predicted that the sensorimotor processing and the congruency effects are altered by glucose. In line with the literature, the results show a typical flanker effect, i.e., participants responded slower and less accurately in incongruent compared to congruent and neutral trials. In contrast to our hypothesis this effect was similar for the placebo and the glucose condition. *Post hoc* analyses further revealed significantly slower RTs in the first block of the glucose condition relative to placebo. This slowing effect was only observed for the first block of trials when glucose was administered in the first session (session effect) and it was equally found for congruent and incongruent trials. These results might be explained by the fact that the slowing effect occurred when participants were relatively inexperienced in the SR mapping. This fits with previous work showing high task difficulty to be one prerequisite for glucose effects on cognition (Kennedy and Scholey, 2000; Riby, 2004). However, in contrast to our hypothesis we found a glucose impairment effect rather than the usual glucose facilitation effect. In addition, we did not observe any influence of flanker congruency on the degree of glucose slowing. Based on these findings we suggest that sensorimotor function may only be impaired by glucose when certain task aspects are difficult, for instance SR mapping. We hypothesized that the slowing effect observed in Experiment 1 is linked to SR mapping strength, and hence conducted a second experiment where the strength of SR mapping was kept consistently low throughout the experiment.

## EXPERIMENT 2

Experiment 2 tested the hypothesis that RT slowing in the glucose condition occurs when SR mapping is low. For this purpose we altered our flanker task to keep SR mapping consistently low throughout the experiment, which was achieved by using novel stimulus sets in each block for which participants had to learn new SR associations. Through this manipulation we simulated the weaker SR mapping present in block 1 of experiment 1 constantly throughout the study. We predicted that in this case the slowing of RTs in the glucose condition would be present in all blocks. Because the glucose effect observed in experiment 1 was only present in participants who had received glucose in the first session, the second experiment used a between-participants design, rather than a repeated measures design, to avoid carry over effects between sessions when comparing glucose and placebo effects.

### METHODS (EXPERIMENT 2)

#### Participants

Twenty four right-handed volunteers (mean age: 20.1 ± 0.7 years; 21 females) with normal or corrected-to-normal vision took part in this study. This experiment was approved by the Ethics Committee of the University of Surrey. All participants gave informed consent and were paid £7.50 per hour.

#### Design and procedure

**FIGURE 4:**
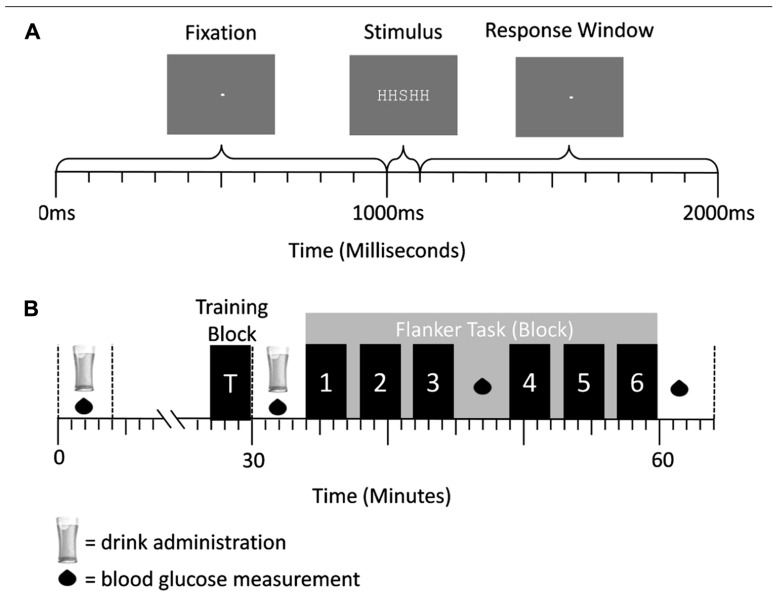
**(A)** Trial structure of the flanker task in experiment 2. A central fixation dot was presented for 1000 ms, followed by an arrow array for 100 ms. Afterward the central fixation dot was presented again for 900 ms. Responses could be conducted after stimulus onset and before the onset of the first fixation cross (response window = 1000 ms). **(B)** Schematic representation of a testing session. Glucose/placebo drinks were administered at 30 min intervals, while BGC measurements were taken every 15 min. The blocks of the flanker task were performed after administration of the second drink. They are represented by the numbered bars.

A double-blind placebo-controlled between-participants design was used where one group of 12 participants received glucose drinks, and the other group of 12 participants had placebo drinks. The experimenter and participant were both blind to the content of the drinks and to the BGC measurements taken during the study. **Figure 4B** provides a schematic representation of the sequence and duration of events during the study. The study protocol was similar to experiment 1, where the breakfast, and glucose/placebo drinks were exactly the same. The only differences were that (1) only behavioral responses were recorded in experiment 2, (2) the flanker task started at 10.00 am, (3) the design of the flanker task changed, and (4) the BGC measurements were taken with a Hemocue 201+ blood glucose meter (Hemocue Ltd.).

#### Flanker task

In this letter flanker task (**Figure 4A**), each trial began with the presentation of a fixation cross for 1000 ms followed by a five-letter stimulus array (e.g., “SSSSS”; array size: 0.5° × 2.5°; similar to (Gratton et al., 1992) for 100 ms. Directly after the letter array the central fixation dot (size: 0.05°) was displayed again for 900 ms, allowing a period of time in which participants could press the response button corresponding to the target. The stimuli appeared on a computer screen in white in the fixed-width font “Courier New” against a gray background under dimmed-light conditions. Participants had a viewing distance of 85 cm. The trial times were exactly the same as in experiment 1.

The five-letter stimulus arrays contained one out of two possible central target letters (e.g., “H” or “S”) to which participants were instructed to respond with the left or right index finger. Participants were asked to ignore the flanking letters. These flanking letters were either congruent (e.g., “SSSSS”) or incongruent to the target, i.e., the letter associated with the opposite response (e.g., “SSHSS”). Within each experimental block each of the four possible target-flanker letter configurations (e.g., HHHHH, SSSSS, SSHSS, and HHSHH) was presented equiprobably and in randomized order.

The experiment consisted of one practice block to familiarize the participant with the general structure of the task, and six sets of blocks consisting of a SR mapping training phase and an associated experimental block. Each of the six set of blocks used a different pair of letters from which stimuli were constructed (”S“ & “H,” “X” & “U,” ” Z” & “D,” “E” & “Y,” “T” & “G,” and “O” & “F”). Stimulus order, i.e., which letter pair was presented in which of the six block sets, and target – response key assignments (e.g., “S” – left key, “H” – right key) were counterbalanced across participants. Each set of blocks lasted 3 min with a 1 min break between them. At the beginning of each set of blocks participants learnt which response corresponded to which central target letter in an SR mapping training phase. This phase required participants to respond correctly in 10 consecutive trials consisting of only a target letter without any flanking stimuli, i.e., five trials with one target letter and five trials of the other target letter, presented in a random order. If participants pressed the wrong response button, responded too late (RT > 1000 ms) or not at all, error feedback was given, i.e., “wrong response” or “too slow,” and the SR mapping training began again. During this phase participants were instructed to respond as accurately as possible. After the SR mapping training phase, participants performed the experimental block which consisted of 80 trials. During this block, participants were instructed to respond as fast and accurately as possible and no error feedback was given. As stated above, another practice block consisting of 12 trials was given at the beginning of the experiment. It was similar to the experimental blocks, except for the following alterations: the letter pair “P” & “K” was used, error feedback was given, and participants were given an accuracy instruction.

#### Data analysis

Blood glucose concentrations were analyzed by comparing the baseline difference between the glucose and placebo condition (1st BGC measurement) with an independent samples *t*-test. Drink effects on the BGC level during the flanker task were assessed with a mixed ANOVA with the between subject factor DRINK TYPE (glucose vs. placebo) and the within-subject factor BGC TIME (2rd–4th BGC measurements). For the flanker task, RTs and choice error rates were separately analyzed in mixed three-way ANOVAs comprising the between-participant factor DRINK TYPE (glucose vs. placebo) and the within-subject factors TRIAL TYPE (congruent vs. incongruent) and BLOCK (1–6). For all ANOVAs, violations of the sphericity were corrected using the Huynh–Feldt correction. *Post hoc* *t*-tests were conducted and Bonferroni corrected as appropriate.

### RESULTS (EXPERIMENT 2)

#### BGC measurements

**FIGURE 5:**
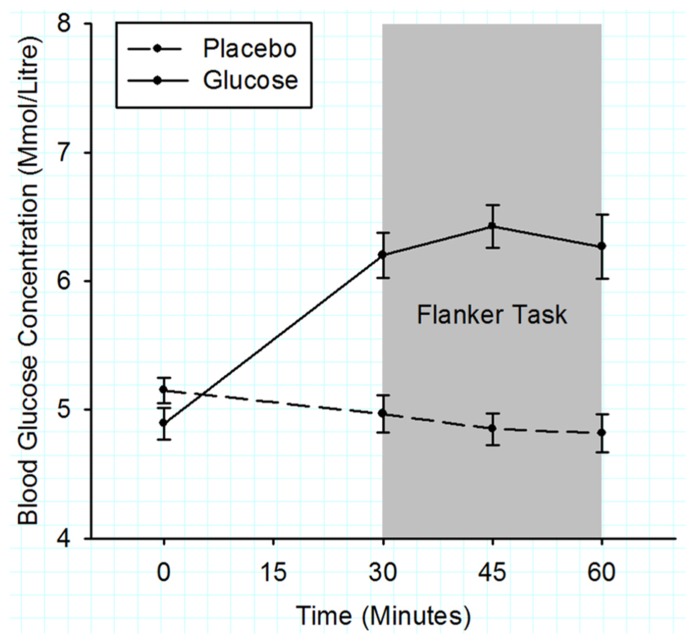
**Mean blood glucose concentrations measured in 15 min intervals for the duration of experiment 2.** Solid lines represent the BGC in the glucose group and dashed lines the BGC in the placebo group. The gray shaded area indicates the time period when the flanker task was performed. Error bars represent the standard error of the mean.

**Figure 5** shows that initial BGC levels were similar for both drink types (placebo = 5.2 ± 0.1 mmol/l, glucose = 4.9 ± 0.1 mmol/l; *t* (22) = 1.63, *p* = 0.12). During the flanker task (2nd–4th BGC measurement), the BGC was significantly elevated in the glucose compared to the placebo condition (glucose = 6.3 ± 0.15 mmol/l, placebo = 4.9 ± 0.15 mmol/l), *F* (1, 22) = 45.3, *p* < 0.001.

#### Behavioral measures

**FIGURE 6:**
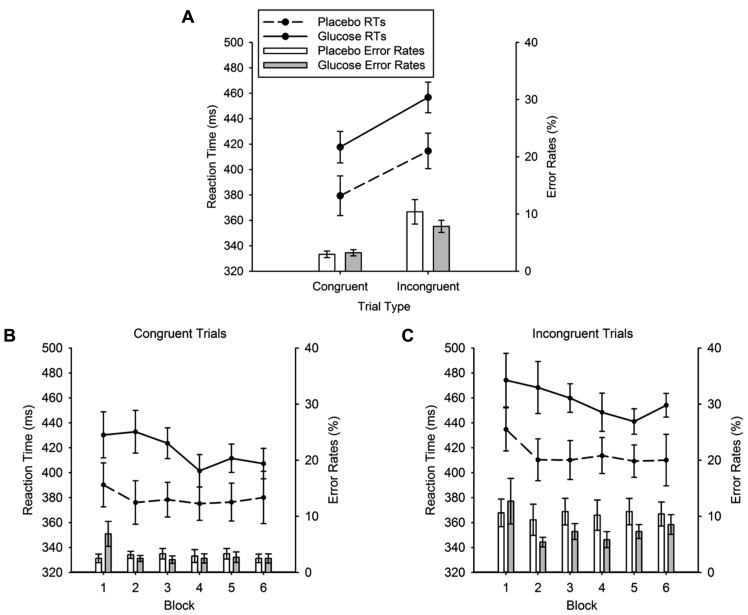
**(A)** Mean reaction times (lines) and error rates (bars) for the congruent and incongruent conditions of the flanker task for glucose (solid lines; gray bars) and placebo (dashed lines, white bars). **(B)** Mean reaction times (lines) and error rates (bars) in the congruent condition, separately for each block. **(C)** Mean reaction times (lines) and error rates (bars) in the incongruent condition, separately for each block. Error bars represent the standard error of the mean.

***Reaction times.*** **Figure 6A** shows the mean RTs for placebo and glucose group for both congruent and incongruent trial types. As expected, responses in incongruent trials were slower than responses in congruent trials (436 ± 9 vs. 398 ± 10 ms). This was reflected in a highly significant main effect of TRIAL TYPE, *F* (1, 22) = 137.98, *p* < 0.001. Critically this analysis revealed that RTs in the glucose group were significantly slower than in the placebo group (glucose = 437 ± 13 ms, placebo = 397 ± 13 ms), yielding a significant effect of DRINK TYPE, *F* (1, 22) = 4.5, *p* = 0.045. The size and direction of the RT difference between placebo and glucose group was similar for the three trial types (congruent: placebo = 379 ± 14 ms, glucose = 418 ± 14 ms; incongruent: placebo = 415 ± 13 ms, glucose = 457 ± 13 ms). This was also evident in the non-significant DRINK TYPE × TRIAL TYPE interaction, *F* < 1. Figures 6B and 6C show that the mean RTs are reduced across successive blocks of the flanker task. This was reflected in a significant main effect of BLOCK, *F* (5, 110) = 2.43, *p* = 0.039. No other main effect or interaction reached significance.

***Error rates.*** The error rates are plotted in **Figure 6A**. Again, the typical flanker congruency effect was found. Errors in the congruent condition were lower than errors in the incongruent condition (3.09 ± 0.39 vs. 9.11 ± 1.19%), which produced a highly significant effect of TRIAL TYPE, *F* = (1, 22) = 40.04, *p* < 0.001. However, there was no main effect of DRINK TYPE (*F* < 1) and no DRINK × TRIAL TYPE interaction, *F* (1, 22) = 2.18, *p* = 0.15. There were also no significant block effects (see Figures 6B and 6C).

### DISCUSSION (EXPERIMENT 2)

The aim of experiment 2 was to replicate the glucose effect observed in block 1 of experiment 1, and to test our main hypothesis that a slowing of RTs is observed for weak SR mapping in the glucose group relative to the placebo group. Confirming this hypothesis, RTs were consistently slowed by 40 ms in the glucose group throughout the task. With regards to response conflict, as indexed by the flanker effect, we could firstly show that our flanker task variant successfully induced a response conflict in the incongruent condition as intended. Both RTs and error rates were increased on incongruent trials relative to congruent trials in both groups. Importantly, the magnitude of this effect was not modulated by drink type. Indeed we demonstrated additivity between drink type and flanker congruency, with average slowing by glucose of 39 ms in congruent trials and 42 ms in incongruent trials. In summary, this pattern of results suggests that sensorimotor processing is slowed by glucose when SR mapping is weak. Critically, response conflict processing was not altered by glucose.

## GENERAL DISCUSSION

The current experiments aimed to investigate the effects of BGC elevation within the normal range, induced by glucose-rich drinks, on the performance of two variants of the Eriksen flanker task (Eriksen and Eriksen, 1974). For both task variants the typical flanker effect was found, with faster and more accurate responses in the congruent compared to the incongruent condition. Moreover, glucose did not alter performance in experiment 1, whereas it had a slowing effect independent of the magnitude of the congruency effect in experiment 2. The main difference between the two experiments was related to the formulation and maintenance of SR mapping rules. In detail, experiment 1 used one set of stimuli throughout the experiments. This meant that SR mapping was strong, and consequently task difficulty was low. In experiment 2, however, novel stimulus sets were introduced in each block, which induced a weaker SR mapping and therefore resulted in a higher task difficulty. Our findings suggest that glucose alters the performance in the flanker task but does so only if SR mapping is weak. The findings also imply that glucose is not necessarily a “cognitive enhancer” as has been found in the majority of previous research (Riby, 2004), but can also weaken performance with regard to response speed. This glucose-induced slowing affects congruent and incongruent trial types equally, and hence does not modulate conflict monitoring per se. These findings will be further discussed and interpreted (1) in line with the assumption of differential effect of glucose on cognitive processes and brain function and (2) in the context of the previous conflict task literature.

### DIFFERENTIAL EFFECTS OF GLUCOSE ON COGNITIVE PROCESSES AND BRAIN FUNCTION

The most striking finding of the current study was that glucose slowed RTs in relation to SR mapping strength but not response conflict, since the congruency effect was similar in both drink conditions. This dissociation between SR mapping strength and conflict monitoring might be explained by the involvement of different brain areas in these processes. More specifically, conflict monitoring is linked to anterior cingulate cortex (ACC) and dorsolateral prefrontal cortex (dlPFC) activation (Bunge et al., 2002; Luks et al., 2010), whereas artificial SR mapping is related to activations in the premotor cortex (Murray et al., 2000), the medial bank of the intraparietal sulcus (Rushworth et al., 2001) and the supramarginal gyrus (Bunge et al., 2002). Moreover, the left inferior parietal sulcus is more active when SR mapping is weak, and this area gradually reduces its activity when SR associations become stronger over time (Grol et al., 2006). This reduction of inferior parietal sulcus activity with strengthening SR associations is most likely due to a transfer for control from the cortex to the basal ganglia, in particular the dorsal striatum (Toni et al., 2001; Atallah et al., 2006; Yin and Knowlton, 2006). Interestingly, similar findings were also reported for the effect of lorazepam and mirtazapine on the performance in a letter flanker task which showed a slowing of RTs without interacting with congruency (de Bruijn et al., 2004). In the context of those findings, we postulate that glucose might primarily affect frontoparietal loops involved in SR mapping, rather than circuitries controlling cognitive control.

This interpretation assumes that glucose does not affect brain function in a uniform manner. This notion is supported by studies showing that glucose it is not utilized uniformly throughout the brain (Sokoloff, 1977; Duelli et al., 1999; Duelli and Kuschinsky, 2001; Harris and Attwell, 2012). Actually, the number and types of glucose-transporters does vary between brain regions (Zeller et al., 1997; Duelli et al., 1999; Choeiri et al., 2002) and some of these transporters are insulin-sensitive, i.e., Glut4, whereas others are not, i.e., Glut3 (Park, 2001; McEwen and Reagan, 2004), which might further enhance differential glucose utilization throughout the brain. In addition, some brain areas might already metabolize glucose close to maximum capacity and hence they do not have the capacity to increase their glucose utilization much further whereas others, like the hippocampal brain regions might not work at optimum level during euglycemia. Therefore the availability of more extracellular glucose strongly improves the performance of those areas (Park, 2001). This is supported by evidence from rat studies which showed that cortical and striatal areas have a higher glucose utilization rate at rest and a smaller increase in glucose utilization with enhanced extracellular glucose levels compared to the hippocampus (Duelli et al., 1999; Duelli and Kuschinsky, 2001). This literature supports the assumption of differential effects of glucose on cognitive processes and brain areas.

### EXPLANATIONS FOR DIFFERENTIAL GLUCOSE EFFECTS ON CONFLICT TASKS

This subsection will discuss the RT slowing effects in the flanker task in relation to the previous reports about glucose effects in sensorimotor conflict tasks. To the best of our knowledge, only four studies investigated glucose effects with a sensorimotor conflict task, i.e., the Stroop task (Benton et al., 1994; Craft et al., 1994; Gailliot et al., 2007; Brown and Riby, 2013), and the current article is the first scientific report of glucose effects on the flanker task, which is another kind of sensorimotor conflict task. Two out of the four studies using the Stroop task implemented a fasting protocol prior to drink administration and task performance (Craft et al., 1994; Brown and Riby, 2013). These studies showed that participants were faster (and made more errors) following glucose in the interference condition compared to baseline condition. This is contrary to the results of the present study.

There are some key methodological differences between these studies and our experiments, which could have contributed to these divergent findings. Firstly, in contrast to the present study, which used an overnight fasting protocol to stabilize the BGC levels and reduce BGC level fluctuations across participants, other studies used no fasting (Benton et al., 1994; Gailliot et al., 2007), or a short 2 h fast prior to the experiment only (Brown and Riby, 2013). Moreover, Brown and Riby (2013) further conducted the experiment between 9 am and 3 pm, while the present experiment was conducted in the morning and hence strictly controlled for variation in the circadian phase. Secondly, the glucose dosage and frequency of drinks was often not the same. More specifically, most studies used one drink with 25–50 g glucose but Benton et al. (1994) gave two drinks (50 and 25 g glucose) to the participants before the task spaced 25 min apart. We had 2/3 drinks with 25 g of glucose that were given to the participants in 30 min intervals in order to keep the BGC levels elevated throughout the experiment. Actually, this might play a crucial role, as Scholey et al. (2001) suggested that glucose facilitation effects are more likely during the falling arm of the blood glucose curve during glucose disposal. In this case, one would expect no glucose facilitation effects when conducting a memory study with our experimental protocol. However, unpublished data from our group show a robust glucose facilitation effects in a verbal declarative memory task with our glucose induction protocol, which suggests that glucose facilitation effects are not necessarily confined to the falling arm of the blood glucose curve.

Methodological differences in glucose dosage, frequency of administration, and task design could be potential contributors to the divergent findings between our study and the Stroop studies mentioned above. However, before coming to this conclusion, it is worth discussing more task-related factors. In addition, it is also true that the cognitive conflict induced by the Stroop task differs quite substantively from the cognitive conflict induced by the flanker task. For example, the Stroop task is performed by inhibiting the processing of conflicting textual information of the color word and by focusing on the task-relevant stimulus feature of ink color. Here the target and distracter information are qualitatively different. In contrast, the distracters in the flanker task have the same physical characteristics as the targets, and can only be distinguished from each other based on their spatial configuration. Several studies support the argument that the Stroop and flanker task should be treated rather differently as they affect, at least partly, other cognitive processes and draw on different sensory domains. This idea is supported by behavior evidence, the RT distributions for the two tasks are not the same and RTs are slower in the Stroop task (Kornblum et al., 1990; Salo et al., 2001; Fan et al., 2003; Tillman and Wiens, 2011). In addition electrophysiological evidence suggests that the conflict-related information is processed at later temporal stages in the Stroop task than in the flanker task (Folstein and Van Petten, 2008; Tillman and Wiens, 2011). Thirdly, fMRI studies have shown that the brain areas and networks activated in these tasks are different although partly overlapping (Fan et al., 2003; Badre et al., 2005).

## CONCLUSION

Previous literature clearly shows that BGC level enhancements within the euglycemic range facilitate cognitive performance, especially in declarative memory and working memory tasks, but also in tasks with high cognitive demands (Riby, 2004). The current study is the first one that employed flanker tasks to study glucose effects on SR mapping and conflict processing. It demonstrated generalized slowing effect which can be specifically linked to weak SR mapping, a frontoparietal network function. This slowing effect occurred to the same degree for congruent and incongruent trials which means that conflict monitoring was not affected by glucose. These findings were discussed in line with the assumption of differential effects of glucose on cognitive processes and brain function, and in relation to previous studies that investigated glucose effects on conflict tasks.

## Conflict of Interest Statement

The authors declare that the research was conducted in the absence of any commercial or financial relationships that could be construed as a potential conflict of interest.

## Footnotes

1 EEG findings will not be reported in this paper.
